# Evidence-Based Second-Line Treatment in RAS Wild-Type/Mutated Metastatic Colorectal Cancer in the Precision Medicine Era

**DOI:** 10.3390/ijms22147717

**Published:** 2021-07-19

**Authors:** Guido Giordano, Pietro Parcesepe, Giuseppina Bruno, Annamaria Piscazzi, Vincenzo Lizzi, Andrea Remo, Massimo Pancione, Mario Rosario D’Andrea, Elena De Santis, Luigi Coppola, Michele Pietrafesa, Alberto Fersini, Antonio Ambrosi, Matteo Landriscina

**Affiliations:** 1 Unit of Medical Oncology and Biomolecular Therapy, Department of Medical and Surgical Sciences, University of Foggia, Policlinico Riuniti, 71122 Foggia, Italy; giuseppina.bruno@unifg.it (G.B.); annamaria.piscazzi@unifg.it (A.P.); 2 Department of Diagnostics and Public Health—Section of Pathology, University and Hospital Trust of Verona, 37134 Verona, Italy; parcesepe.pietro@gmail.com; 3 General Surgey Unit, Policlinico Riuniti, 71122 Foggia, Italy; vincenzo.lizzijr@gmail.com; 4 Pathology Unit “Mater Salutis” Hospital, ULSS9, Legnago, 37045 Verona, Italy; remino76@yahoo.it; 5 Department of Sciences and Technologies, University of Sannio, 82100 Benevento, Italy; massimo.pancione@unisannio.it; 6 UOSD Oncologia, Ospedale S. Paolo, 00053 Civitavecchia, Italy; mariorosario.dandrea@aslroma4.it; 7 Department of Anatomical, Histological, Forensic Medicine and Orthopedic Sciences, Sapienza University of Rome, 00185 Rome, Italy; elena.desantis@uniroma1.it; 8 UOC Anatomia ed Istologia Patologica e Citologia Diagnostica, Dipartimento dei Servizi Diagnostici e della Farmaceutica, Ospedale Sandro Pertini, ASL Roma 2, 00157 Roma, Italy; luigi.coppola@aslroma2.it; 9 Laboratory of Pre-Clinical and Translational Research, IRCCS, Referral Cancer Center of Basilicata (CROB), Rionero in Vulture, 85028 Potenza, Italy; michele.pietrafesa@crob.it; 10 General Surgery Unit, Department of Medical and Surgical Sciences, University of Foggia, Policlinico Riuniti, 71122 Foggia, Italy; alberto.fersini@unifg.it (A.F.); antonio.ambrosi@unifg.it (A.A.)

**Keywords:** colorectal cancer, second-line therapy, precision medicine, chemotherapy, anti-angiogenic treatment, anti-EGFR, immunotherapy, RAS, BRAF, MSI

## Abstract

Target-oriented agents improve metastatic colorectal cancer (mCRC) survival in combination with chemotherapy. However, the majority of patients experience disease progression after first-line treatment and are eligible for second-line approaches. In such a context, antiangiogenic and anti-Epidermal Growth Factor Receptor (EGFR) agents as well as immune checkpoint inhibitors have been approved as second-line options, and RAS and BRAF mutations and microsatellite status represent the molecular drivers that guide therapeutic choices. Patients harboring K- and N-RAS mutations are not eligible for anti-EGFR treatments, and bevacizumab is the only antiangiogenic agent that improves survival in combination with chemotherapy in first-line, regardless of RAS mutational status. Thus, the choice of an appropriate therapy after the progression to a bevacizumab or an EGFR-based first-line treatment should be evaluated according to the patient and disease characteristics and treatment aims. The continuation of bevacizumab beyond progression or its substitution with another anti-angiogenic agents has been shown to increase survival, whereas anti-EGFR monoclonals represent an option in RAS wild-type patients. In addition, specific molecular subgroups, such as BRAF-mutated and Microsatellite Instability-High (MSI-H) mCRCs represent aggressive malignancies that are poorly responsive to standard therapies and deserve targeted approaches. This review provides a critical overview about the state of the art in mCRC second-line treatment and discusses sequential strategies according to key molecular biomarkers.

## 1. Introduction

Colorectal cancer (CRC) represents the third most common cancer worldwide and the second cause of cancer-related death [1,2]. About 20% of patients with CRC have advanced disease at time of diagnosis, and 35% of patients treated with curative intent will develop metastatic disease [1,2].

During the last two decades, a great improvement in the cure and the prognosis of metastatic CRC (mCRC) has been observed, due to the advances in molecular biology in addition to the introduction of active chemotherapy and target-oriented agents [3,4]. The molecular characterization of human CRC has been object of extensive investigation in the past years and the Cancer Genome Atlas Network pointed out that, excluding the hypermutated cancers, CRCs are characterized by considerably similar patterns of genomic alterations. Twenty-four genes were found to be significantly mutated, and most of them belong to three major signaling pathways: Wnt and TGFβ, PI3K and RTK/RAS, and p53 [5].

In particular, two pathways have been investigated in a clinical perspective: the Epidermal Growth Factor Receptor (EGFR) and the Vascular Endothelial Growth Factor-(VEGF) cascades [6]. The anti-EGFR agents, cetuximab and panitumumab were shown to increase the overall survival (OS) in phase III randomized trials with anticancer activity limited to the *RAS* wild-type mCRC population [7,8,9]. In fact, mutations in *RAS* genes predict a lack of response to these agents in either first- or second-line settings [10]. Consistently, RAS mutations have been related to a worse prognosis in mCRC patients [10].

Moreover, the anti-VEGF molecules bevacizumab, aflibercept, and ramucirumab have improved mCRC outcomes in several phase III studies [11,12,13]. It is universally accepted that the exposure of mCRC patients to all active drugs during the clinical course of the disease prolongs survival; this represents a strong rationale for sequential therapeutic strategies [14]. Chemotherapy schedules containing fluoropyrimidines, oxaliplatin, and irinotecan (either in doublet or in triplet combinations) together with targeted agents (cetuximab, panitumumab, or bevacizumab), depending on *RAS* mutational status, represent the standard of care in mCRC first-line treatment [15,16].

Recent evidence suggests that the prognosis of mCRCs and the activity of first-line regimens is also affected by the primary tumor site, with the right-side mCRCs characterized by a worst prognosis and *RAS* wild-type mCRCs arising in left colon being more sensitive to EGFR-based therapy compared to *RAS* wild-type mCRCs arising in right colon [17,18,19]. *BRAF* mutations are found in 8–12% of cancers, with *BRAF^V600E^* accounting for more than 90% of mutations in *BRAF*-mutated cancers [20,21]. It is known that *BRAF* mutations are responsible for worse prognosis and therapy resistance, representing, at the same time, a potential target for new drugs and combinations [22,23,24].

In fact, recent data from a phase III trial demonstrated longer survival in *BRAF* mutant mCRC patients treated with combination of three agents, Encorafenib, Binimetinib, and Cetuximab compared to chemotherapy plus cetuximab [25]. Furthermore, a high tumor mutation burden has emerged as a marker of responsiveness to immunotherapy in several cancers [26,27]. In 2017, based on practice-changing phase I-II data, the US FDA approved the immune checkpoint inhibitors, pembrolizumab and nivolumab as second-line treatments of mCRCs that are mismatch-repair-deficient (dMMR) or have high levels of microsatellite instability (MSI-H) [28,29,30]. By contrast, immune checkpoint inhibitors are ineffective in mismatch-repair-proficient (pMMR), microsatellite-stable (MSS), and low levels of microsatellite instability (MSI-L) tumors [28].

The choice of second-line schedule depends on the previous regimen, *RAS*/*BRAF*/MSI status, treatment aim, and patient profile [31]. Evidence from randomized phase III studies indicated that the continuous inhibition of tumor angiogenesis with bevacizumab, aflibercept, or ramucirumab beyond the first progression to bevacizumab may improve survival [32]. In addition, anti-angiogenic agents showed interesting second-line activity in combination with chemotherapy in patients treated with anti EGFR agents in first-line.

Fewer data are available regarding the use of anti-EGFR drugs beyond progression to an anti-EGFR based first-line therapy; thus, second-line anti-EGFR monoclonals are generally used in *RAS* wild-type tumors after the failure of first-line bevacizumab-based therapy [30,33]. Emerging data are available in *BRAF* mutated and MSI-H mCRC, and novel strategies have been recently added to the Oncologist’s portfolio of drugs in the management of these patients.

Finally, as defined by gene expression profiling approaches, mCRC has been divided into four distinct consensus molecular subtypes (CMSs). The four subtypes are CMS1, with MSI-H and immune activation (14%); CMS2, with canonical CRC alterations (37%); CMS3, with metabolic dysregulation (13%); and CMS4, with mesenchymal features (23%) [22,34]. Those subtypes reflect distinct biological states and have been shown to be both prognostic for OS and predictive for benefit from cetuximab and bevacizumab in the CALGB 80405 and AIO-FIRE3 trials.

Patients classified as CMS1 appear to derive more benefit from bevacizumab, while those classified CMS2 appear to derive more benefit from cetuximab [35,36]. Therefore, in this intricate scenario, a therapeutic sequential strategy using the most effective combinations of chemotherapy and target agents both in first- and second-line settings is desirable to maximize patient outcomes. In this article, we present all second-line therapeutic options for mCRC, showing the mechanism of action of each molecule and phase III trial data. Furthermore, we discuss the potential rationale supporting the use of sequential strategies, using the available agents after first-line therapy.

## 2. Antiangiogenic Drugs in mCRC Second-Line Therapy

Currently, bevacizumab, aflibercept, and ramucirumab are the anti-angiogenic drugs approved by regulatory authorities in mCRC second-line therapy, in combination with standard chemotherapy. The mechanisms of action and clinical indication of these molecules are summarized in Table 1.

### 2.1. Bevacizumab

Bevacizumab is a humanized monoclonal antibody binding VEGF-A, which is able to prevent the interaction with its receptor Vascular Endothelial Growth Factor Receptor-2 (VEGFR-2) [37]. It represents a cornerstone in mCRC first-line therapy, in combination with irinotecan-, oxaliplatin-, and fluoropyrimidine-based doublet and triplet schedules [11,38,39,40]. Notably, the role of bevacizumab has been investigated in mCRC second-line treatment after failure of either anti-EGFR or bevacizumab-based regimens. The ECOG 3200 study randomly assigned 829 patients to receive either FOLFOX 4 plus bevacizumab, FOLFOX 4 or bevacizumab alone as second-line therapy for mCRC in patients previously treated with irinotecan- and fluoropyrimidine-based regimens for advanced disease.

The OS was significantly longer in the FOLFOX 4 plus bevacizumab arm compared to FOLFOX 4 and single agent bevacizumab (Table 2). Major grade 3–4 toxicities in the FOLFOX 4 plus Bevacizumab arm were represented by hypertension, bleeding, neuropathy, and vomiting [41]. Two observational studies (BRITE and ARIES) suggested that the use of post-progression bevacizumab may result in better outcomes [42,43]. Based on these results, a prospective, intergroup, randomized, open-label, phase III study (ML18147) was designed.

Patients with mCRC, Eastern Cooperative Oncology Group (ECOG) Performance status (PS) 0–2, who had progressed within 4 weeks to a bevacizumab plus standard first-line chemotherapy (flouropyrimidine plus either oxaliplatin or irinotecan) and who were not candidates for primary metastasectomy were included in this trial. At the time of enrolment, patients were randomized to receive treatment with fluoropyrimidines plus irinotecan or oxaliplatin with or without bevacizumab. The second-line regimen was chosen according to the first-line schedule (i.e., patients who received first-line irinotecan were switched to second-line oxaliplatin and vice versa).

The primary end-point was OS; the secondary end-points were Progression Free Survival (PFS), OS from the start of first-line treatment, response rate (RR), and safety. Eight-hundred-twenty patients were randomly assigned to bevacizumab plus chemotherapy (*n* = 409) or chemotherapy alone (*n* = 410) with an intention-to-treat population of 819 patients. The median OS was superior in the bevacizumab plus chemotherapy treatment arm compared to chemotherapy alone.

Consistently, the median PFS was significantly longer in the bevacizumab beyond progression versus chemotherapy alone group. The response rate (RR) was similar in the two groups (Table 2), whereas a higher disease control rate (DCR) was obtained in the bevacizumab arm compared to chemotherapy alone (68% vs. 54% respectively; *p* < 0.0001) in a post hoc analysis.

The median OS from the start of the first-line treatment was retrospectively documented, and no differences were observed between bevacizumab plus chemotherapy (23.9 months) and chemotherapy alone (22.5 months; *p* = 0.17). Safety analysis indicated no substantial differences between the two study groups [44]. A very similar study (BEBYP trial) was performed in 19 Italian centers; however, the announcement of ML18147 results led to its premature interruption. A partial results publication furnished data consistently aligned with the ML18147 trial findings [45].

### 2.2. Aflibercept

Aflibercept is a humanized recombinant fusion protein that binds to VEGF-A, VEGF-B, and Placental Growth Factor (PlGF), thus, inhibiting interaction with their specific receptors [46]. This agent acts as a decoy receptor deriving from the fusion of VEGFR-1 and VEGFR-2 extracellular domains with Fc part of human IgG1 [46]. The particular technology and structure allow a more complete angiogenesis blockade than other anti-angiogenic agents, also influencing the cross talk between tumor microenvironment and pro-angiogenic factors [47].

VELOUR was an international, double-blinded, phase III trial in which 1226 patients with mCRC, who had progressed to oxaliplatin-based first-line treatment, were randomized to receive either combination of FOLFIRI plus aflibercept or FOLFIRI plus placebo. The primary end-point of this study was the OS; secondary end-points were PFS, RR, and safety. Notably, patients early relapsed during oxaliplatin-based adjuvant chemotherapy, as well as bevacizumab pre-treated patients were included in this study. FOLFIRI plus aflibercept significantly increased the median OS compared to FOLFIRI plus placebo; PFS was also longer in the aflibercept plus FOLFIRI group.

The addition of aflibercept to FOLFIRI almost doubled the RR respect of FOLFIRI plus placebo (Table 2). Major grade 3–4 toxicities in the aflibercept arm were diarrhea (19.3%), hypertension (19.3%), stomatitis and ulcerations (13.7%), neutropenia (36.7%), asthenic condition (16.9%), infections (12.3%), thromboembolic events (9.7%), proteinuria (7.9%) and gastrointestinal perforation (0.5%) [12]. A further analysis by Ruff et al. observed a progressive increase in OS difference between the two study groups over the course of time. In this analysis, a 4.4-month difference in favor of aflibercept-based treatment at 24 months was demonstrated [48].

The efficacy of aflibercept-based treatment was maintained also in elderly patients (≥65 years), and it was independent from prior bevacizumab treatment in a predefined analysis [49,50]. The benefit of aflibercept plus FOLFIRI schedule was also consistent in further post hoc analysis evaluating the time of progression from first-line therapy (<9 months versus ≥9 months) in oxaliplatin plus bevacizumab pre-treated patients [51]. In a post hoc analysis excluding patients that recurred during or within 6 months of completing adjuvant treatment, the gain in OS within aflibercept arm increased from 1.4 to 1.9 months [52].

The median OS from the start of first-line treatment until death was significantly higher in aflibercept plus FOLFIRI versus placebo plus FOLFIRI group (25.95 versus 22.87 months, respectively), whereas rapid relapsers were excluded [53]. Chau et al. attempted to identify the better efficacy subgroup in the VELOUR ITT population by using prognostic factors. Patients with no fast relapse from adjuvant treatment, ECOG PS 0, and any number of metastatic sites or ECOG PS 1 with <2 metastatic sites showed better OS, PFS, and RR [54]. Notably, a biomarker analysis performed in the VELOUR study population evaluated the role of the mutational status of *K-RAS* exon 2, extended *RAS*, and *BRAF* in order to identify subgroups of patients with differential treatment effects.

None of the mutation subgroups showed a significant interaction; in particular, a major benefit was demonstrated in *BRAF* mutant patients with the addition of aflibercept. The same analysis showed that tumor sidedness did not affect the aflibercept efficacy [55]. Following the VELOUR results, the Aflibercept Safety and Quality of Life Program (ASQoP) international, multicenter, open-label, single arm phase IIIb/IV study was performed. Safety of the treatment was the primary end-point, and health-related quality of life was the secondary end-point. Globally, this study showed a similar safety profile of aflibercept plus FOLFIRI combination in a real-life population [56].

### 2.3. Ramucirumab

Ramucirumab is a fully human IgG1 monoclonal antibody that binds the VEGFR-2 extracellular domain preventing the interaction between all VEGF ligands and their receptor [57]. This drug represents another option in second-line therapy for mCRC patients who had progressed to a bevacizumab plus oxaliplatin first-line treatment [58]. The international, multicenter, double-blinded, randomized, phase III study RAISE evaluated the efficacy of second-line ramucirumab plus FOLFIRI versus placebo plus FOLFIRI in mCRC.

One-thousand-seventy-two patients with ECOG PS 0–1, known K-RAS exon 2 mutations, progressed during or within 6 months from the last dose of first-line combination therapy of bevacizumab plus oxaliplatin and fluoropyrimidines were enrolled. Patients with poorly controlled hypertension, thromboembolic events within 12 months form randomization, or during first-line therapy and grade 3–4 bleeding or proteinuria events during first-line treatment were excluded. Region, K-RAS mutation status, and time to disease progression after starting first-line treatment were considered as stratification factors.

The OS was the primary study end-point; PFS, RR, DCR, safety, and quality of life evaluation were secondary end-points. Both median OS and PFS were superior in the ramucirumab plus FOLFIRI versus placebo plus FOLFIRI group. No differences in RR were observed in the two study groups (Table 2). Major grade 3–4 toxicities in the experimental arm were diarrhea (11%), fatigue (12%), neutropenia (38%), bleeding/hemorrhage (3%), and hypertension (12%). Despite that this study was not statistically powered for subgroup analysis, the ramucirumab benefit was consistent in all subgroups [13].

A pre-specified analysis showed that *K-RAS* wild-type patients had a significant OS increase in the ramucirumab plus FOLFIRI arm compared to placebo plus FOLFIRI (14.4 versus 11.9 months respectively; HR 0.82; *p* = 0.049). Conversely, a smaller but not statistically significant benefit was observed in *K-RAS* mutant patients. The correlation between first-line time to progression (TTP) and ramucirumab benefit was also investigated, and no OS benefit in <6 months first-line TTP patients was observed. Patients who had ≥6 months first-line TTP achieved a longer but not significant OS in the ramucirumab arm versus control (14.3 versus 12.5 months, respectively).

These results may be influenced by the under-power of this study for subgroups evaluation. Age-based analysis (<65 versus ≥65) showed a similar survival advantage with ramucirumab in both groups [59]. Post hoc analyses of RAISE patients evaluated the association of *RAS/RAF* mutational status and the site of the primary tumor (left versus right) with efficacy parameters. Notably, the ramucirumab plus FOLFIRI schedule improved patient outcomes, regardless of the *RAS/RAF* mutational status and the primary tumor site.

Ramucirumab treatment provided a numerically substantial, although not statistically significant, benefit in *BRAF*-mutated tumors, possibly due to the small sample of *BRAF* mutated patients [60]. Further exploratory post-hoc analyses of RAISE data showed that patients with treatment-emergent neutropenia had longer OS compared with those without and patients with low versus high baseline absolute neutrophil count also had longer OS [61]. Interestingly, the survival benefit was independent from the baseline CEA levels (high > 10 vs. low ≤ 10) [62].

## 3. Anti-EGFR Drugs in mCRC Second-Line Treatment

Two monoclonal antibodies (i.e., cetuximab and panitumumab) have been approved in mCRC second-line treatment with the limitation to *RAS* wild-type patients. The mechanisms of action and clinical indications of these molecules are shown in Table 1.

### 3.1. Cetuximab

Cetuximab is a chimeric IgG1 monoclonal antibody that targets the extracellular domain of the EGFR and blocks ligand-induced tyrosine-kinase downstream signaling. This molecule also exerts its effect by its immunomodulatory activity via antibody-dependent cell-mediated cytotoxicity (ADCC). Cetuximab stimulates ADCC activity when its constant region (Fc) binds to a Natural Killer cell receptor (CD16/*FcγRIIIa*) leading their own lytic activity on tumor cells [63]. The combination of cetuximab and chemotherapy represents a standard of care in mCRC first-line treatment in *RAS* wild-type patients according to the results of phase III randomized trials, especially in tumors arising in the left colon [7,19,64].

The role of cetuximab has been also investigated in second-line mCRC therapy. The BOND phase III study randomized 329 mCRC refractory patients, who had progressed to at least one prior therapy to receive either cetuximab plus irinotecan or cetuximab alone. Combination treatment showed significant benefit in PFS and RR, with no differences in OS (Table 2) [65]. Second-line cetuximab in combination with irinotecan in refractory mCRC patients also showed a significantly higher PFS and response rate compared to irinotecan alone in the EPIC phase III randomized trial. No difference in the median OS was observed between the two study arms (Table 2) [66]. Both in BOND and EPIC studies, most relevant grade 3–4 toxicities related to Cetuximab treatment were represented by skin rash, diarrhea, hypomagnesemia, and associated electrolyte imbalance.

The role of cetuximab treatment beyond the progression to cetuximab-based first-line therapy was investigated in a phase II study. The CAPRI trial evaluated 340 *K-RAS* wild-type patients who had received first-line therapy with FOLFIRI plus cetuximab. At the time of progression, 153 patients were randomized to receive second-line treatment with either FOLFOX plus cetuximab or FOLFOX alone. In the ITT population, the median PFS was 6.4 versus 4.5 months in FOLFOX plus cetuximab and FOLFOX-alone arms, respectively (HR 0.81, 95% CI 0.58–1.12; *p* = 0.19).

Accordingly, no significant difference in the median OS between the two study groups was observed (17.6 vs. 14.0 in combination regimen and FOLFOX alone, respectively; HR 0.86, 95% CI 0.61–1.20; *p* = 0.41). RR in the FOLFOX plus cetuximab arm was 21.6% vs. 12.7% in FOLFOX group. A further Next Generation Sequencing (NGS) retrospective analysis on 22 genes involved in EGFR-pathway was performed in order to evaluate the impact of *K-N-RAS*, *BRAF* or *PIK3CA* mutations on patient outcomes.

NGS analysis was done in 117 of 153 patients, and a trend in better outcomes (PFS, OS and RR) was observed in *K-N-RAS*, *BRAF*, and *PIK3CA* quadruple wild-type patients treated with FOLFOX plus cetuximab. A detrimental effect of FOLFOX plus cetuximab was observed in patients harboring at last one mutation in these genes. Continuation of cetuximab beyond first progression was not associated with an increased toxicity profile [67].

### 3.2. Panitumumab

Panitumumab is a fully human monoclonal IgG2 antibody that binds to the extracellular domain of EGFR inhibiting ligand-induced downstream signaling [68]. A combination of panitumumab and chemotherapy in *RAS* wild-type populations is a standard of care in mCRC first-line therapy [9]. A randomized phase III trial evaluated the benefit of adding panitumumab to FOLFIRI versus FOLFIRI alone in 1186 mCRC patients who had progressed to a fluoropyrimidine-based first-line therapy. Patients were prospectively tested and analyzed for *K-RAS* mutational status. OS and PFS were the co-primary study end points in *K-RAS* wild-type and mutant patients, respectively.

No differences in OS and PFS were observed in *K-RAS* mutant population within the two study arms, without detrimental effects. Panitumumab plus FOLFIRI significantly increased PFS versus FOLFIRI alone but not OS in *K-RAS* wild-type patients. In this population, RR was higher in panitumumab plus FOLFIRI arm compared to FOLFIRI alone (Table 2) [69]. The combination of panitumumab and irinotecan in mCRC patients progressed to a first-line fluoropyrimidine therapy was also investigated in the PICCOLO trial.

In this study, 1198 mCRC patients without molecular selection were randomly allocated in panitumumab plus irinotecan or irinotecan alone treatment arms. The study protocol was amended in order to allow stratification according to *K-RAS* mutational status. The final analysis was performed in 460 *K-RAS* wild-type patients previously untreated with anti-EGFR molecules. The addition of panitumumab to irinotecan showed a significant improvement in PFS and RR versus irinotecan alone (Table 2) [70]. The panitumumab toxicity profile was characterized by a higher incidence of skin rash and diarrhea.

## 4. Braf Inhibitors in mCRC

The *BRAF^V600E^* mutation is the more common *BRAF* mutation in CRC and is responsible for a poor prognosis, resulting in nearly a two-fold increase in mortality relative to wild-type *BRAF* in the metastatic setting [71]. Usually, *BRAF^V600E^* mutations are associated with a right-sided primary tumor, advanced age, female sex, high tumor grade, and precursor sessile serrated adenomas [72]. *BRAF^V600E^* CRC is also associated with the CpG island methylator phenotype (CIMP status), which may result in the epigenetic inactivation of *MLH1*, inducing a mismatch repair deficiency (dMMR) and, consequently, a MSI phenotype [73].

Among *BRAF^V600E^* mCRC patients, approximately 20% exhibit deficient dMMR [72]. More than 200 *BRAF* uncommon (*non-V600E*) mutations have been identified, with a combined incidence ranging from 1.6% to 5.1%. The prognosis of these patients appears to be similar to those with wild-type *BRAF* mCRC [74]. Although *BRAF* inhibitors have been practice-changing in the treatment of *BRAF*-mutated melanoma, they demonstrated a surprising and striking lack of efficacy as single agents in patients with colorectal cancer harboring *BRAF^V600E^* mutations [75,76].

The knowledge that the biology is more complex and heterogeneous in CRC than in melanoma is supported by several preclinical data suggesting that, differently from melanoma, CRC cell lines express high levels of activated EGFR, which convey a reactivation of MAPK pathway following *BRAF* inhibition [77,78]. In preclinical studies, anti-EGFR therapy with either small molecule inhibitors (i.e., erlotinib) or monoclonal antibodies (i.e., cetuximab) rendered cell lines sensitive to the *BRAF* inhibitors. Furthermore, there are additional pathways involved in the resistance to *BRAF* inhibitors in *BRAF*-mutated CRC cells and, among others, the PI3K/AKT signaling pathway and the crosstalk between Wnt and MAPK pathways [79,80].

These preclinical data suggest that both EGFR activation and aberrant PI3K signaling as well as interaction with the Wnt pathway may underlie the limited therapeutic activity of *BRAF* inhibitor monotherapy in patients with *BRAF*-mutated mCRC [81]. Therefore, combining drugs targeting both MAPK and Wnt pathways may also be an effective strategy in managing *BRAF*-mutated mCRC [82,83]. One of the major strategies investigated in the last few years is represented by the combination of *BRAF* inhibitors and anti-EGFR antibodies.

Fifteen patients with *BRAF^V600E^* mCRC were treated with vemurafenib (BRAF inhibitor) and panitumumab, and 10 of 12 evaluable patients showed some tumor regression with two patients showing a partial response and stable disease lasting more than 6 months [84]. Thus, a newer combination treatment, including the addition of targeted agents to chemotherapy, was subsequently evaluated. The combination of irinotecan and cetuximab with or without vemurafenib was studied in 106 patients with *BRAF^V600E^* mCRC in a phase II study (SWOG 1406). This study demonstrated that the triplet regimen was characterized by better PFS and ORR (17% versus 4%; *p* = 0.05) with a disease control rate of 65% versus 21% [85].

Preclinical models showed that the pharmacological inhibition of *BRAF* leads to the suppression of ERK-dependent negative feedback mediators that, in turn, results in RAS and other RAF kinase activation. Consequently, the combination of *BRAF* and MEK inhibitors may overcome this mechanism of resistance, resulting in a strong suppression of this pathway. In a phase I/II clinical trial, 142 mCRC patients were enrolled into three different treatment arms, including a triplet arm containing dabrafenib (*BRAF* inhibitor), trametinib (MEK inhibitor), and panitumumab (EGFR inhibitor), as well as two doublet arms of panitumumab with either dabrafenib or trametinib.

The confirmed response rate was 21% in the triplet arm and 10% in the panitumumab/dabrafenib arm. No response was observed in the panitumumab/trametinib arm. This study confirmed the activity of targeted treatments for *BRAF^V600E^* CRC and the relevance to obtain a simultaneous and more potent inhibition on MAPK axis [86]. Encorafenib is a highly selective ATP-competitive small molecule RAF kinase inhibitor, which suppresses the *RAF/MEK/ERK* pathway in tumor cells expressing the *BRAF^V600E^* mutation (Table 1).

As for vemurafenib, no responses were observed with encorafenib monotherapy [87]. Promising results were observed in a dose-escalation trial with encorafenib and cetuximab in 26 *BRAF*-mutated CRC patients. The RR was 23.1%, 14 patients achieved stable disease, the clinical benefit rate was 54%, and the median PFS was 3.7 months [88]. In the following phase II trial, 102 mCRC patients who had received at least two prior therapies were randomized to receive encorafenib plus cetuximab with or without the *PIK3A* inhibitor alpelisib.

A pre-specified PFS analysis comparing the triplet to the doublet showed a median PFS of 5.4 and 4.2 months and confirmed ORR of 27% and 22%, respectively. Interim OS analysis (triplet vs. doublet) showed a median OS of 15.2 months with the triplet combination, while the OS was not reached in patients receiving the doublet [89].

The BEACON study is a phase III trial in 665 patients with *BRAF^V600E^* metastatic CRC who have received more than one line of prior treatment and that were randomized (1:1:1) to a triplet arm of encorafenib, binimetinib (MEK inhibitor), and cetuximab, an encorafenib and cetuximab arm, or an investigator choice arm of either irinotecan/cetuximab or FOLFIRI/cetuximab. The study included a safety lead-in phase with triplet treatment prior to initiation of the randomized component of the study. Thirty patients were enrolled in the safety lead-in phase, the overall RR was 48%, and the median PFS and OS were 8.0 months and 15.3 months, respectively.

The triplet regimen was well tolerated with five dose-limiting toxicities observed (two serous retinopathy, one reversible left ventricular ejection fraction decrease, and two cetuximab-related infusion reactions) [90]. Given the very encouraging preliminary response data, this regimen was granted a Breakthrough Therapy designation by the U.S. FDA and was also added to the NCCN guidelines as a first-line treatment option for *BRAF^V600E^* CRC. At the final analysis of this trial, the primary endpoints were reached, with a median OS of 9.0 months in the triplet-therapy group and 5.4 months in the control group, with ORR of 26% and 2%, respectively.

Patients who received the doublet therapy achieved a median OS of 8.4 months and ORR of 20%, with a risk reduction of death of 40% compared with the control group. Consistently, PFS was longer in patients receiving triplet (4.3 months) or doublet therapy (4.2 months), compared with the control group (1.5 months)—Table 2. Overall, grade 3 to 4 adverse events were observed in 58% of patients in the triplet-therapy group, 50% of patients in the doublet-therapy group, and 61% of patients in the control group.

As expected, the MEK tyrosine kinase inhibitor-class toxicities were reported in patients treated with the triplet-therapy but with a low incidence. The most common toxicities in the encorafenib plus binimetinib plus cetuximab arm were represented by gastrointestinal-related and skin-related events, including diarrhea, nausea, vomiting, and acneiform dermatitis. Low hemoglobin level or anemia was a common laboratory abnormality [25].

According to the updated results from the BEACON CRC trial, triplet and doublet therapy confirmed their superiority over standard chemotherapy, showing a median OS of 9.3 for both the triplet and the doublet regimens versus 5.9 months for the standard chemotherapy regimen. In addition, a longer maintenance of quality of life was observed in patients treated in the encorafenib plus cetuximab and the binimetinib plus encorafenib plus cetuximab arms compared to those who received the standard chemotherapy.

Interestingly, there were no significant differences in the median time to deterioration in quality of life according to the two chemotherapy-free treatment groups [91,92]. Based on these results, on June 2020, the European Medicines Agency (EMA) approved the combination of encorafenib and cetuximab in adult patients with mCRCs harboring the *BRAF^V600E^* mutation who had received prior systemic therapy.

## 5. Immune-Checkpoint Inhibitors in mCRC

CRC can be categorized into two discrete subgroups based on the mutational burden: tumors that have a dMMR–MSI-H status and a high mutational burden (>12 mutations per 106 DNA bases) and tumors that have a pMMR–MSI-L status with a much lower mutational burden (<8.24 mutations per 106 DNA bases) [93]. Defective DNA mismatch repair can be detected either by the lack of immunohistochemical staining of the MMR proteins (i.e., MLH1, MSH2, MSH6, or PMS2) or by PCR-identified alterations in the lengths of microsatellites between the tumor and the corresponding normal tissue or blood.

MSI-H is the hallmark of tumors in patients with Lynch syndrome; however, the development of dMMR–MSI-H is a sporadic event owing to somatic defects in MMR gene function, most commonly the hypermethylation of the MLH1 promoter. Importantly, dMMR–MSI-H tumors are heavily infiltrated by immune cells, i.e., CD8+ tumor-infiltrating lymphocytes (TILs), T helper 1 (TH1) CD4+ TILS, and macrophages, and have a microenvironment that is rich in type I interferons in comparison with other CRCs [94,95]. Approximately 15% of all CRCs are dMMR–MSI-H [96].

The presence of dMMR–MSI-H disease is prognostic, as stage 2 dMMR–MSI-H tumors have a lower risk of recurrence than stage 2 pMMR–MSI-L ones [97]. Stage 4 dMMR–MSI-H tumors constitute only 2–4% of all mCRCs. Patients with dMMR–MSI-H mCRC have a dismal prognosis, but the expression of PD1, PDL1, and CTLA4 is substantially upregulated in their cancers [28,98,99]. These observations suggested that dMMR–MSI-H CRCs might respond well to an immune checkpoint blockade. In initial studies published between 2010 and 2013, immune checkpoint inhibitors demonstrated very limited clinical activity in non-selected CRC.

Nivolumab, an anti-PD1 antibody (Table 1), was evaluated in 19 patients, and, initially, no responses were reported; however, one patient, with a dMMR–MSI-H status, had a response at 21 months, and, after retreatment, this patient achieved a complete response that lasted ≥3 years [100,101]. A phase II trial of the anti-PD1 antibody pembrolizumab (Table 1) was reported in 2015, in which three separate cohorts of patients were treated: dMMR–MSI-H mCRCs, pMMR–MSI-L mCRCs, and dMMR–MSI-H non-CRCs. Of the 10 patients with dMMR–MSI-H CRC, four had a partial response and five had a stable disease at 20 weeks.

At that time point, the median PFS and OS were not yet reached in the dMMR–MSI-H cohort but were 2.2 months and 5.0 months, respectively, in the pMMR–MSI-L cohort [28]. In updated results, the response rate was 50% (95% CI 31–69%), and the disease control rate was 89% in the 28 patients with dMMR–MSI-H tumors. At 24 months, PFS was 61%, and overall survival was 66%. None of the 18 patients with pMMR–MSI-L CRC responded. This study demonstrated the benefit of immune checkpoint blockade in dMMR–MSI-H tumors [102,103] (Table 2).

In CheckMate 142 trial, another PD1 inhibitor, nivolumab, was tested in 74 patients with dMMR–MSI-H mCRC. At a median follow-up of 12 months, 31% of patients achieved an investigator-assessed objective response, and 69% of patients a disease control for ≥12 weeks. The median PFS was 14.3 months, the 12-months PFS was 50% and the 12 months-OS was 73% [104]. The combination of nivolumab with ipilimumab was also evaluated in this trial. Among the 30 patients enrolled, 9 patients (33%) achieved an objective response, and 14 patients (52%) achieved disease stabilization [105].

The updated results of the CheckMate 142 trial in the complete cohort of 119 patients demonstrated an objective response rate of 55% and a tumor burden reduction from the baseline in 77% of patients. At that time point, the median PFS was not yet reached, and the 9-month and 12-month PFS results were 76% and 71%, respectively. The median OS was not reached, and the 9-month and 12-month OS results were 87% and 85%, respectively.

Treatment with combined nivolumab and ipilimumab resulted in an increased rate of drug-related immune-related adverse events: 32% of patients experienced grade 3–4 treatment-related toxicities compared to 20% of patients treated with nivolumab alone [106,107] (Table 2). On the basis of the compelling data in dMMR–MSI-H CRCs, the FDA granted accelerated approval to pembrolizumab in May 2017 and to nivolumab in July 2017 for the second-line treatment of patients with dMMR–MSI-H CRC.

Recently, practice-changing data of a phase III randomized study (KEYNOTE-177 trial) were published. This trial was conducted at 192 sites in 23 countries. Patients (*n* = 307) with MSI-H mCRC were randomly assigned in a 1:1 ratio to first-line pembrolizumab at a dose of 200 mg every 3 weeks or to the investigator’s choice of chemotherapy. The choices of chemotherapy were as follows: mFOLFOX6, mFOLFOX6 plus bevacizumab; mFOLFOX6 plus cetuximab; FOLFIRI, FOLFIRI plus bevacizumab; or FOLFIRI plus cetuximab.

Treatment was continued for a maximum of 35 treatments with pembrolizumab or until disease progression, development of unacceptable toxic effects, illness, or a decision from the physician or patient to withdraw from the trial. At the second interim analysis, after a median follow-up of 32.4 months, pembrolizumab was superior to chemotherapy with respect to PFS (median, 16.5 vs. 8.2 months; hazard ratio, 0.60; *p* = 0.0002). The estimated restricted mean survival after 24 months of follow-up was 13.7 months (range 12.0 to 15.4) as compared with 10.8 months (range 9.4 to 12.2). Data on the overall survival are still evolving (66% of required events had occurred) and remain blinded until the final analysis.

An overall response was observed in 43.8% of the patients in the pembrolizumab group and 33.1% in the chemotherapy group. Among patients with an overall response, 83% in the pembrolizumab group, as compared with 35% of patients in the chemotherapy group, had ongoing responses at 24 months. Treatment-related adverse events of grade 3 or higher occurred in 22% of the patients in the pembrolizumab group, as compared with 66% (including one patient who died) in the chemotherapy group.

Immune-mediated adverse events and infusion reactions occurred in 47 patients (31%) in the pembrolizumab group as compared with 18 (13%) in the chemotherapy group. Grade 3 or 4 events of interest occurred in 14 patients (9%) and 3 patients (2%), respectively, with colitis (3%) and hepatitis (3%) most common in the pembrolizumab group [108].

## 6. Sequential Second-Line Strategies in Metastatic CRCs

Current evidence shows that second-line therapies may have a positive impact on mCRC survival [109]. Therefore, it is important to identify therapeutic strategies, in order to (i) optimize the use of available drugs, maximizing long-term efficacy, (ii) reduce toxicities, and (iii) assure a better quality of life to mCRC patients. There are no strong biomarkers that may directly influence therapeutic second-line choices. Therefore, treatment strategies should be based on patient-related factors (age, PS, comorbidity, and preferences) and disease-related factors (tumor aggressiveness, disease burden, presence of symptoms, *RAS/BRAF* mutational status, and MSI-H). However, a major driver in second-line decision making is the regimen used in first-line. Thus, in the subsequent paragraphs, alternative strategies will be discussed starting from the option used as the first-line therapy.

### 6.1. Second-Line after Progression to Beavacizumab-Based First-Line Treatment

First-line bevacizumab-based therapy represents the standard of care in *RAS* mutant mCRCs independently form the site of primary tumor and, more recently, has been proposed as more effective strategy in right-side mCRCs independently from *RAS* mutational status, based on the evidence that right-side, *KRAS* wild-type tumors have a lower benefit with first-line anti-EGFR agents [17,19]. Different therapeutic options are available today at the time of disease progression after bevacizumab-based first-line treatment, and they depend on *RAS* mutational status.

**Figure 1:**
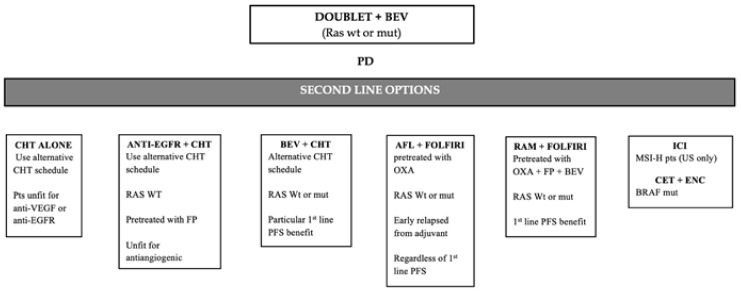
Second-line therapeutic options after disease progression to a doublet + Bevacizumab first line therapy. PD: progression disease; BEV: Bevacizumab; CHT: chemotherapy; wt: wild-type; mut: mutant; IRI: Irinotecan; FP: fluoropyrimidines; AFL: Aflibercept; OXA: Oxaliplatin; RAM: Ramucirumab; FP: fluoropyrimidines; ICI: immune-checkpoint inhibitors; US: United States; CET: Cetuximab; ENC: Encorafenib.

In *RAS* wild-type patients not pre-treated with an anti-EGFR antibody (mostly right side mCRCs), the combination of chemotherapy with cetuximab or panitumumab could represent an option (Figure 1). Notably, there are no clinical phase III studies showing a clear and significant OS advantage for anti-EGFR drugs in this setting. Nevertheless, American (NCCN) and European (ESMO) Guidelines for mCRC management recommend of anti-EGFR drugs in combination with chemotherapy in *RAS* wild-type patients progressed to bevacizumab first-line treatment [15,16].

Moreover, ESMO Guidelines underline that second-line OS benefits for anti-EGFR agents are comparable with those achieved in later lines. Conversely, the Italian Association for Medical Oncology (AIOM) Guidelines do not recommend the use of anti-EGFR agents as second-line therapy [110]. In this scenario, there is lack of data about the use of anti-EGFR molecules in combination with oxaliplatin in second-line treatment. In fact, phase III studies with cetuximab and panitumumab have been performed with irinotecan-based schedules (Table 2).

Alternatively, chemotherapy alone or in combination with antiangiogenic molecules represents an available option both in *RAS* wild-type non-candidate for anti-EGFR drugs and *RAS* mutant patients. In particular, chemotherapy alone could be reserved for those patients who experienced limiting toxicities during bevacizumab treatment or patients unfit for combination therapy with doublets plus target agents (Figure 1). In such a context, a major issue is the selection of the second-line antiangiogenic agent (bevacizumab beyond progression or aflibercept/ramucirumab) in patients progressing first-line bevacizumab-based therapy.

The current knowledge from clinical trials suggests that mCRC patients may benefit of sustained angiogenesis inhibition also beyond first progression [111]. Use of continuative anti-VEGF treatments might induce less resistance to chemotherapy by creating a suitable environment for genetically stable endothelial cells [112]. The use of bevacizumab plus chemotherapy beyond first progression is supported by a biological background, and the results of the ML18147 trial showed an OS and PFS advantage compared to chemotherapy alone (Table 2).

The advantage in survival was maintained independently of age, ECOG PS, first-line chemotherapy schedule, first-line PFS (≤ or >9 months), and *KRAS* mutations. Nevertheless, no differences in response rate were observed, and the OS calculated from the beginning of first-line treatment was similar in the two study arms. The ML18147 trial excluded patients with first-line PFS of less than 3 months as well as patients receiving less than 3 consecutive months of first-line bevacizumab. Exploratory subgroup analysis suggested a major benefit for bevacizumab beyond-progression in patients with first-line PFS > 9 months (HR 0.73, 95% CI: 0.58–0.92).

Differently, the subgroup treated with bevacizumab beyond progression with a first-line PFS ≤ 9 months showed only a trend toward a better OS (HR 0.89, 95% CI: 0.73–1.09) [41]. These results suggest that the use of bevacizumab beyond first progression could be a valid therapeutic option in patients with slowly progressive disease who received a significant benefit during bevacizumab-based first-line therapy (Figure 1).

Sustained VEGF inhibition induces resistance through activation of different pro-angiogenic ligands like PlGF and VEGF-D that are increased following progression on bevacizumab treatment [113,114]. Consistently, PlGF shares structural homology with VEGF-A, but stimulates angiogenesis via interaction with VEGFR-1 [115]. These mechanisms support the use of alternative antiangiogenic molecules, like aflibercept or ramucirumab, after progression to a bevacizumab first-line therapy (Figure 1). Aflibercept is characterized by a peculiar mechanism of action that allows a wider spectrum of angiogenesis blockade and may help to overcome resistances to a bevacizumab first-line treatment [47].

The VELOUR phase III trial showed that second-line combination of FOLFIRI plus aflibercept increased OS, PFS, and RR compared to FOLFIRI plus placebo in mCRC patients pre-treated with oxaliplatin. Patients enrolled in the VELOUR study were “less-selected” with respect to the ML18147 trial patients. The VELOUR population included first-line “early progressors” (PFS < 3 months), patients relapsed within 6 months of completing oxaliplatin-based adjuvant therapy, and patients having <3 consecutive months of first-line bevacizumab. Only 30% of VELOUR patients were pre-treated with bevacizumab, and a pre-specified subgroup analysis confirmed the survival advantage for the aflibercept arm regardless of prior bevacizumab exposure.

A further post hoc analysis, in the bevacizumab pre-treated population, showed that FOLFIRI plus aflibercept was superior to FOLFIRI plus placebo in terms of OS and PFS independently of first-line PFS (≤ or >9 months). The use of aflibercept showed benefit regardless of *RAS* and *BRAF* mutational status. Therefore, aflibercept after progression to a bevacizumab-based first-line therapy could be used in rapid as well as slow “progressors”, in patients who need tumor shrinkage or symptoms control, despite the age and *RAS* mutational status, with the limitation to oxaliplatin pre-treated patients (Figure 1).

Finally, the opportunity to shift from bevacizumab to aflibercept was also supported by a biomarker analysis performed on the plasma samples of VELOUR patients. Nine biomarkers implicated in angiogenesis or bevacizumab resistance were tested on 553 patients of whom 169 had received prior bevacizumab. VEGF-A and PlGF were significantly increased in bevacizumab-pretreated patients (five-fold and two-fold higher levels in the prior bevacizumab versus no prior bevacizumab group, respectively) [116,117].

The latest drug that could be employed in bevacizumab pre-treated patients is ramucirumab, which targets angiogenesis by blocking VEGFR-2 and preventing the interaction of all VEGF ligands and the consequent receptor activation [57]. The RAISE phase III trial confirmed a significant improvement of median OS and PFS without an effect on RR with the combination of FOLFIRI plus ramucirumab compared to FOLFIRI plus placebo in patients who had received oxaliplatin, fluoropyrimidines, and bevacizumab as first-line treatment.

Ramucirumab plus FOLFIRI was shown to improve outcomes regardless of age, ECOG PS, or *KRAS* status. Patients with <6 months TTP during first-line therapy received less benefit from this combination. However, the OS and PFS gain observed in the RAISE study appeared to be not different from bevacizumab or aflibercept in the same setting. Interestingly, serum biomarkers analysis carried out in the RAISE study patients, identified high VEGF-D level as a potential, predictive biomarker of response to FOLFIRI + Ramucirumab second line treatment [118].

Ramucirumab plus FOLFIRI is available in the United States, while in Europe it has been approved by European Medicine Agency, but it is not reimbursed in all the countries. The cost-to-effectiveness ratio represents an important issue to consider at the time of second-line therapeutic choice and should be incorporated into the decision-making process. In the context of mCRC antiangiogenic second-line options, ramucirumab has showed a survival benefit similar to bevacizumab and aflibercept but has a higher cost [119].

### 6.2. Second-Line Therapy in Ras Wild-Type Tumors Treated with Anti-Egfr First-Line Therapy

**Figure 2:**
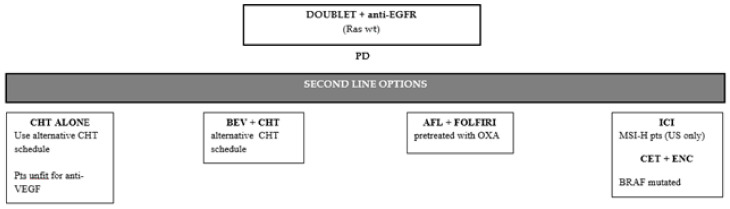
Second-line therapeutic options after disease progression to a doublet + anti-EGFR first line therapy. PD: progression disease; BEV: Bevacizumab; CHT: chemotherapy; wt: wild-type; FP: fluoropyrimidines; AFL: Aflibercept; OXA: Oxaliplatin; FP: fluoropyrimidines; ICI: immune-checkpoint inhibitors; US: United States; CET: Cetuximab; ENC: Encorafenib.

First-line cetuximab- or panitumumab-based therapy is the gold standard in *RAS* wild-type mCRCs despite the site of the primary tumors, even though the magnitude of its activity is stronger in tumors arising in the left colon [17,120]. In such a context, the selection of the second-line therapy after failure of anti-EGFR monoclonals in first-line does not represent a major issue (Figure 2). Indeed, data supporting use of EGFR inhibitors beyond progression are very limited and, consequently, international guidelines uniformly support second-line anti-angiogenic agents [15,16].

As reported in previous paragraphs, either bevaicizumab or aflibercept and ramucirumab combined with chemotherapy were shown to provide clinical benefit in *RAS* wild-type mCRCs treated with first-line anti-EGFR agents [12,13,44]. Unfortunately, no selection criteria are available to choose the best antiangionenic agent in this setting. Therefore, the major drivers in decision making are the backbone chemotherapy used in first-line (i.e., Ramucirumab is allowed only in patients pretreated with oxaliplatin and bevacizumab; aflibercept is allowed only in patients pretreated wit oxaliplatin), the toxicity profile of antiangiogenic agents, and patient-related factors, such as age, comorbidity, and preferences.

### 6.3. Second-Line Therapy after First-Line Triplet Chemotherapy Plus Bevacizumab

The randomized phase III TRIBE study enrolled 508 patients with untreated mCRCs and showed that the combination of triplet chemotherapy (FOLFOXIRI) combined with bevacizumab significantly improved the RR (65% versus 53%, *p* = 0.006), PFS (12.1 months versus 9.7 months, HR 0.75, *p* = 0.003), and OS (29.8 months versus 25.8 months, HR 0.80, *p* = 0.030) compared to FOLFIRI plus bevacizumab [40]. Thus, FOLFOXIRI plus bevacizumab is currently regarded as a standard first-line option in mCRCs, especially as a neoadjuvant treatment in fit patients with initially unresectable diseases or in patients with good ECOG PS and a poor prognosis tumor (i.e., right colon or *BRAF*-mutated tumors).

A major concern regarding FOLFOXIRI-based therapy is whether the up-front combination of all three cytotoxic agents may limit disease control with second-line treatments. Indeed, literature data suggest that initial FOLFOXIRI treatment does not impair the possibility to obtain further objective responses and delay tumor progression with second-line treatments containing the same agents used in first-line. In TRIBE trial, second-line treatments were administered in 166 patients in the FOLFOXIRI plus bevacizumab arm, 23% of them receiving an oxaliplatin-containing second-line treatment (7% oxaliplatin doublet plus bevacizumab, 7% FOLFOXIRI plus bevacizumab, 8% oxaliplatin doublet alone, and 1% FOLFOXIRI alone).

Bevacizumab beyond disease progression was administered to 30% of patients in combination with FOLFOXIRI (8% of cases) or with doublet oxaliplatin- or irinotecan-based chemotherapy. Thirty-one percent of patients received an anti-epidermal growth factor receptor monoclonal antibody as second-line treatment in combination with irinotecan-based doublet. Extremely intriguing is the evidence supporting the re-challenge of FOLFOXIRI in second-line setting after first-line triplet chemotherapy. Recently, a phase III, randomized TRIBE-2 trial, investigated two different approaches with bevacizumab beyond progression.

This study randomized 679 mCRC patients to receive either first-line FOLFOX-6 plus bevacizumab followed by second-line FOLFIRI plus bevacizumab or first-line FOLFOXIRI plus bevacizumab followed by reintroduction of the same regimen after progression. The primary endpoint of the study was PFS2, defined as the time from randomization to disease progression on any treatment given after first disease progression or death. Data from this trial suggest a significant advantage by upfront FOLFOXIRI plus bevacizumab in terms of PFS2 (19.1 vs. 16.4 mos, HR 0.74, 95% CI 0.62–0.88, *p* < 0.001), RR (62% vs. 50%, OR 1.61, 95% CI 1.19–2.18, *p* = 0.002) and first-PFS (12.0 vs. 9.8 mos, HR 0.75, 95% CI 0.63–0.88, *p* < 0.001).

**Figure 3:**
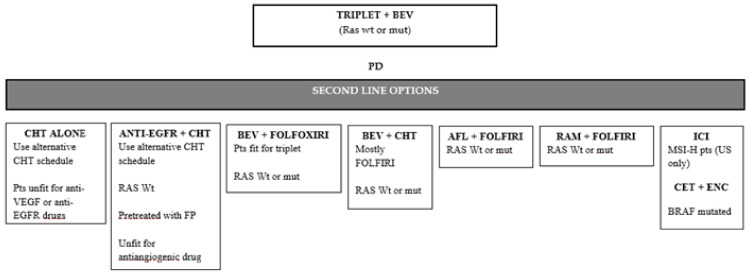
Second-line therapeutic options after disease progression to a triplet + Bevacizumab first line therapy. PD: progression disease; BEV: Bevacizumab; CHT: chemotherapy; wt: wild-type; mut: mutant; IRI: Irinotecan; FP: fluoropyrimidines; AFL: Aflibercept; OXA: Oxaliplatin; RAM: Ramucirumab; FP: fluoropyrimidines; ICI: immune-checkpoint inhibitors; US: United States; CET: Cetuximab; ENC: Encorafenib.

A significant OS benefit for patients in the experimental arm was also observed (27.6 vs. 22.6 mos, HR: 0.81, 95% CI: 0.67–0.98, *p* = 0.033) [121,122]. Altogether, these data support the use of upfront FOLFOXIRI plus bevacizumab. Thus, in this setting, appropriate second-line options include the reintroduction of the same agents after disease progression or doublet chemotherapy regimens combined with bevacizumab or anti-EGFR monoclonals depending on *RAS* mutational status, patient comorbidity, and first-line toxicities (Figure 3).

## 7. Current Strategies and Future Perspectives

Over the last decades, mCRC patient survival increased from 6 months in the absence of active schedules to more than 30 months using the most effective combinations of chemotherapy and target agents. In this landscape, it has been demonstrated that the use of all active drugs is key to maximizing the benefits and prolonging survival. The continuous evolution of mCRC molecular characterization as well as the identification of prognostic and predictive biomarkers also led clinicians to more tailored therapeutic choices. Moreover, the introduction of new regimens/agents in second-line treatment has made more challenging the individualization of a correct sequential strategy.

Continuing angiogenesis suppression beyond first progression or the introduction of an antiangiogenic agent after first-line anti-EGFR monocolonals are, at present, optimal options in second-line settings. Less convincing is the clinical benefit obtained by anti-EGFR agents in *RAS* wild-type mCRCs (mostly right side tumors) treated with first-line bevacizumab-based therapy. In the absence of head-to-head clinical trials between second-line options and biomarkers predictive for response to antiangiogenic drugs, the capability of medical oncologists to select an appropriate therapy is currently based on (a) the patient profile and preferences; (b) disease characteristics; (c) treatment aims; (d) safety profile; and (e) cost-effectiveness.

The recent introduction of upfront triplet chemotherapy combined with bevacizumab represents another challenge for the rational selection of second-line therapy. However, increasing data suggest that upfront triplet chemotherapy does not limit the possibility to obtain disease control in second-line upon reintroduction of the same regimen or doublet chemotherapy plus a target agent.

In the perspective to optimize therapy selection and improve patient outcomes, the recent data obtained in specific molecular subgroups are extremely relevant, i.e., *BRAF*-mutated or MSI-H mCRCs. *BRAF*-mutated mCRCs represent a cohort of patients with poor prognosis and limited effective therapeutic options. While the selection of the appropriate sequential strategy in these patients is still controversial, the recent data in favor of the combination of BRAF inhibitors and anti-EGFR agents are extremely promising.

Consistently, the outbreak of MSI-H as a predictive biomarker of response to immune-checkpoint inhibitors opens the perspective to identify a further subgroup of mCRC patients that does not benefit from first-line chemotherapy plus targeted agents but rather from anti-PD1 agents. While there are no data in support of a sequential therapeutic strategy in MSI-H mCRCs, doublet/triplet chemotherapy plus biological agents are, at present, the standard second-line therapy in this setting.

Finally, treatment personalization and rationale sequential therapy design require the clinical characterization of novel predictive biomarkers. In such a context, novel actionable targets are under preclinical/clinical validation with the aim to further customize therapeutic options and develop novel systemic agents targeting specific oncogenic pathways. *HER2* amplification, *NTRK* rearrangements, Wnt signaling, and *POLE* mutations, among others, represent promising targets to develop novel effective anticancer agents [123,124,125,126].

Consistently, several studies have investigated new potential biomarkers of response to cetuximab in patients harboring *KRAS* mutations. In particular, subjects carrying the non-functional receptor KIR2DS4 showed longer OS compared with carriers of the full-length variant. Similarly, a higher disease control rate was described in KRAS-mutated patients carrying the *FcγRIIa* H131 allele [127].

Furthermore, epigenomics with DNA methylation profiling and transcriptomics represent innovative strategies to characterize novel predictive/prognostic signatures. In such a context, several studies are ongoing with the aim to evaluate the usefulness of patient gene expression profiles and CMS subtypes in treatment choice [128]. Similarly, DNA methylation biomarkers for outcome prediction and treatment decision are still under validation in CRC, based on the preliminary evidence that the hypermethylation of specific subsets of genes may predict poor outcomes and the likelihood of response to specific therapeutic strategies.

## Figures and Tables

**Table 1:** Target agents approved in mCRC second-line treatment.

Drug	Type of Molecule	Mechanism of Action	2nd Line Labelling
Bevacizumab	Humanized MoAb	Binding VEGF-A	Plus standard CHT
Aflibercept	Recombinant Fusion Protein	VEGFR decoy binding VEGF-A/B; PlGF	Plus FOLFIRI in OXA pretreated
Ramucirumab	Fully Human MoAb	Binding VEGFR2	Plus FOLFIRI in OXA + FP + BEV pretreated
Cetuximab	Human-Mouse Chimeric MoAb	Binding EGFR	Plus IRI in *RAS* wt
Panitumumab	Fully Human MoAb	Binding EGFR	Plus FOLFIRI in *RAS* wt
Encorafenib	Threonine and Serine kinase Inhibitor	*BRAF* gene Inhibition	Plus CET in *BRAF^V600E^* mt
Pembrolizumab	Humanized MoAb	Binding PD-1	MSI-H or dMMR FP, OXA and IRI pretreated
Nivolumab *	Fully Human MoAb	Binding PD-1	MSI-H or dMMR FP, OXA and IRI pretreated

MoAb: Monoclonal Antibody; CHT: chemotherapy; OXA: oxaliplatin; FP: fluoropyrimidines; BEV: bevacizumab; wt: wild type; MSI-H: microsatellite instability-high; dMMR: mismatch repair deficient; and * approved alone or plus ipilimumab.

**Table 2:** mCRC second-line phase II and phase III pivotal trials.

Study [Ref.]	Pts	Study Arms	RR (%)	PFS (Months)	HR (95% CI)	OS (Months)	HR (95% CI)
E3200 [41]	829	FOLFOX4 + BEV vs. FOLFOX4 vs. BEV	22.7 vs. 8.6 vs. 3.3 *p* < 0.0001	7.3 vs. 4.7 vs. 2.7 *p* < 0.0001	0.61 (NA)	12.9 vs. 10.8 vs. 10.2 *p* < 0.0011	0.75 (NA)
ML18147 [44]	820	BEV + CHT vs. CHT alone	6 vs. 4	5.7 vs. 4.1 *p* < 0.0001	0.68 (0.59–0.78)	11.2 vs. 9.8 *p* < 0.0062	0.81 (0·69–0·94)
BEBYP [45]	185	CHT + BEV vs. CHT	21 vs. 17 *p* = 0.124	6.8 vs. 5.0 *p* = 0.010	0.70 (0.52–0.95)	15.5 vs. 14.1 *p* = 0.043	0.77 (0.56–1.06)
VELOUR [12]	1226	AFL + FOLFIRI vs. PBO + FOLFIRI	19.8 vs. 11.1 *p* < 0.0001	6.90 vs. 4.67 *p* < 0.0001	0.758 (0.661–0.869)	13.50 vs. 12.06 *p* < 0.0032	0.817 (0.713–0.937)
RAISE [13]	1072	RAM + FOLFIRI vs. PBO + FOLFIRI	13.4 vs. 12.5 *p* = 0.63	5.7 vs. 4.5 *p* = 0.0005	0.793 (0.697–0.903)	11.7 vs. 13.3 *p* < 0.0219	0.844 (0.730–0.976)
EPIC [66]	1298	IRI + CET vs. IRI	16.4 vs. 4.2 *p* = 0.0001	4 vs. 2.6 *p* = 0.0001	0.692 (0.617–0.776)	10.7 vs. 10 *p* = 0.71	0.975 (0.854–1.114)
BOND [65] °	329	IRI + CET vs. CET	22.9 vs. 10.8 *p* = 0.007	4.1 vs. 1.5 *p* = 0.001	0.54 (0.42–0.71)	8.6 vs. 6.9 *p* = 0.48	0.91 (0.68–1.21)
STUDY 181 [69]	1186	FOLFIRI + PAN vs. FOLFIRI	35 vs. 10 § *p* < 0.0001	5.9 vs. 3.9 § *p* = 0.004	0.73 (0.59–0.90)	14.5 vs. 12.5 § *p* = 0.12	0.85 (0.70–1.04)
PICCOLO [70]	460ç	IRI + PAN vs. IRI	34 vs. 12 *p* < 0.0001	not available ^ *p* = 0.015	0.78 (0.65–0.95)	10.9 vs. 10.4 *p* = 0.91	1.01 (0.83–1.23)
BEACON [25]	665	ENC + BIN + CET vs. ENC + CET vs. FOLFIRI + CET	26 vs. 20 vs. 2 *p* < 0.001	4.3 vs. 4.2 vs. 1.5 *p* < 0.001	0.40 * (0.31–0.52)	9.0 vs. 8.4 vs. 5.4 *p* < 0.001	0.60 * (0.45–0.79)
KEYNOTE ** 164 [102,103]	124	PEMBRO	33	4.1	NA	31.4	NA
CHECKMATE 142 ** [106]	119	NIVO + IPI	55	NR	NA	NR	NA

Pts: patients; BEV: Bevacizumab; CHT: Chemotherapy; AFL: Aflibercept; PBO: Placebo; RAM: Ramucirumab; CET: Cetuximab; IRI: Irinotecan; PAN: Panitumumab; ENC: Encorafenib; BIN: Binimetinib; PEMBRO: Pembrolizumab; NIVO: Nivolumab; IPI: Ipilimumab. ° Number of previous treatments (%): one line: (20.7%), two lines (36.5%), and three lines (42.9%); § Only wild type *KRAS* population; Ç Subgroup of patients with *KRAS* c.12–13, 61 wild-type tumors and no previous EGFR targeted therapy; ^ Median PFS in months is not available; * HR for the doublet arm; NA: not available; NR: not reached; and **: Phase II non-randomized studies.

## Data Availability

Not applicable.
